# Integration of single-cell RNA sequencing and spatial transcriptomics to reveal the glioblastoma heterogeneity

**DOI:** 10.12688/f1000research.126243.2

**Published:** 2023-08-09

**Authors:** Adrian Perdyan, Urszula Lawrynowicz, Monika Horbacz, Bozena Kaminska, Jakub Mieczkowski

**Affiliations:** 1 3P-Medicine Laboratory, Medical University of Gdansk, Gdansk, Poland; 2 Department of Medical Immunology, Medical University of Gdansk, Gdansk, Poland; 3 Nencki Institute, Warszawa, Poland

**Keywords:** single-cell RNA sequencing, spatial transcriptomics, glioblastoma, heterogeneity, immunology, sex

## Abstract

Glioblastoma (GBM), a deadly brain tumor, is still one of a few lasting challenges of contemporary oncology. Current therapies fail to significantly improve patient survival due to GBM tremendous genetic, transcriptomic, immunological, and sex-dependent heterogeneity. Over the years, clinical differences between males and females were characterized. For instance, higher incidence of GBM in males or distinct responses to cancer chemotherapy and immunotherapy between males and females have been noted. Despite the introduction of single-cell RNA sequencing and spatial transcriptomics, these differences were not further investigated as studies were focused only on revealing the general picture of GBM heterogeneity. Hence, in this mini-review, we summarized the current state of knowledge on GBM heterogeneity revealed by single-cell RNA sequencing and spatial transcriptomics with regard to genetics, immunology, and sex-dependent differences. Additionally, we highlighted future research directions which would fill the gap of knowledge on the impact of patient’s sex on the disease outcome.

## Introduction

**Figure 1.**
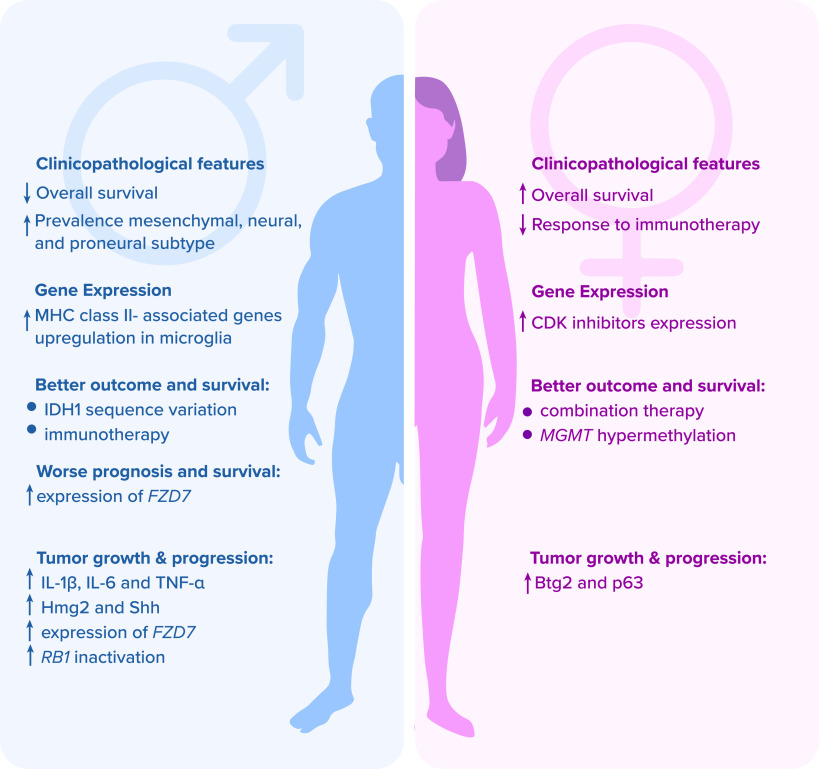
Major sex-dependent clinicopathological and gene expression differences affecting therapeutic outcomes, prognosis, genetics, and immunology of glioblastoma patients. Legend: *Btg2* – B cell translocation gene 2; *CDK* – cyclin-dependent kinase; *FZD7* – frizzled class receptor; Hmg2 – high mobility group box 2; IDH1 – isocitrate dehydrogenase 1; IL – interleukin; MGMT – O6-methylguanine-DNA methyltransferase; MHC – major histocompatibility complex; p63 – transformation-related protein 63; RB1 – retinoblastoma protein 1; Shh – sonic hedgehog human; TNF-α – tumor necrosis factor alpha.

Glioblastoma (GBM) is one of the deadliest human tumors, with a 14-month median survival length and five-year overall survival (OS) of approximately 6.8% ( https://seer.cancer.gov/). Despite intensive research and the introduction of novel therapeutic regimens, the survival rate has not been improved in the last decades. ^1^ The disappointing results are mainly due to ineffective surgical resection and rapid local progression. ^2^ Moreover, there are no useful biomarkers to detect the emergence of GBM, and the early course of the disease is often asymptomatic. ^3^ Nowadays, GBM is treated with surgery, temozolomide-based (TMZ) chemotherapy, and radiotherapy. ^2^ The failure of conventional and targeted therapies is most likely due to intratumoral heterogeneity, intrinsic mechanisms of cell death resistance (due to a high frequency of *TP53* and *PTEN* mutations) and redundant prosurvival signaling pathways. ^4^ Additionally, in accordance with recent reports, patient sex may have a major impact on GBM therapeutic outcomes and prognosis. The response rate to conventional therapies is higher in females, whereas immunotherapy works better in males due to higher molecular and cellular heterogeneity of glioma cells. ^5^ Moreover, a mutation in the isocitrate dehydrogenase 1 encoding gene, *IDH1*, contributes to better chemotherapy outcomes and prolonged OS in males only. ^6^^–^^8^ On the other hand, hypermethylation of the promoter of the *MGMT* gene coding for O6-methylguanine-DNA methyltransferase enhances the effect of TMZ chemotherapy and prolongs OS in females. ^8^^,^^9^ Also, there are several negative prognostic biomarkers which are listed in the Figure 1. ^8^^,^^10^^–^^13^

Over ten years ago, the first GBM transcriptional subtypes were identified with a partial enrichment of *PDGFRA* or *EGFR* alterations. ^14^^,^^15^ However, with the recent introduction of single-cell RNA sequencing (scRNA-seq) and spatial transcriptomics (ST), new cellular states were revealed. Interestingly, distinct states may co-exist within the tumor with variate frequency and are associated with genetic alterations. ^4^^,^^16^ Moreover, GBM heterogeneity manifests in unique developmental states of GBM cells in the tumor. GBM mimics mechanisms of neural development, thus it contains GBM stem cells (GSCs) which are thought to play a major role in possessing tumor-propagating potential and exhibiting preferential resistance to radiotherapy and chemotherapy. ^17^^,^^18^ Finally, GBM has a heterogeneous and highly immunosuppressive TME composed of normal brain residents such as neurons, astrocytes, oligodendrocytes, and microglia, immune system infiltrating cells including mostly monocytes/macrophages, as well as endothelial and mesenchymal cells. ^19^^,^^20^ Glioma-associated microglia and macrophages (GAM) are the most abundant population of immune cells, constituting up to 30% of the tumor mass. ^21^^–^^23^ Despite the high content of glioma-associated microglia and macrophages (GAM), GBM is considered immunologically “cold” due to relatively poor infiltration of activated T cells and anergic state of those present in TME. ^24^ Moreover, the broad range of cell-to-cell interactions between cancer cells and components of the TME affects the biological status of the tumor with an increase in its evasion capacity and resistance to treatment. ^25^

In summary, a better understanding of multilayer GBM heterogeneity, including genetics, epigenetics, developmental stages, TME, and immunology, is required to establish effective therapies. ^16^ Thus, the purpose of this mini-review was to discuss recent advances in exposing the GBM heterogeneity and highlight future research directions.

### Integration of single-cell RNA sequencing and spatial transcriptomics

**Figure 2.**
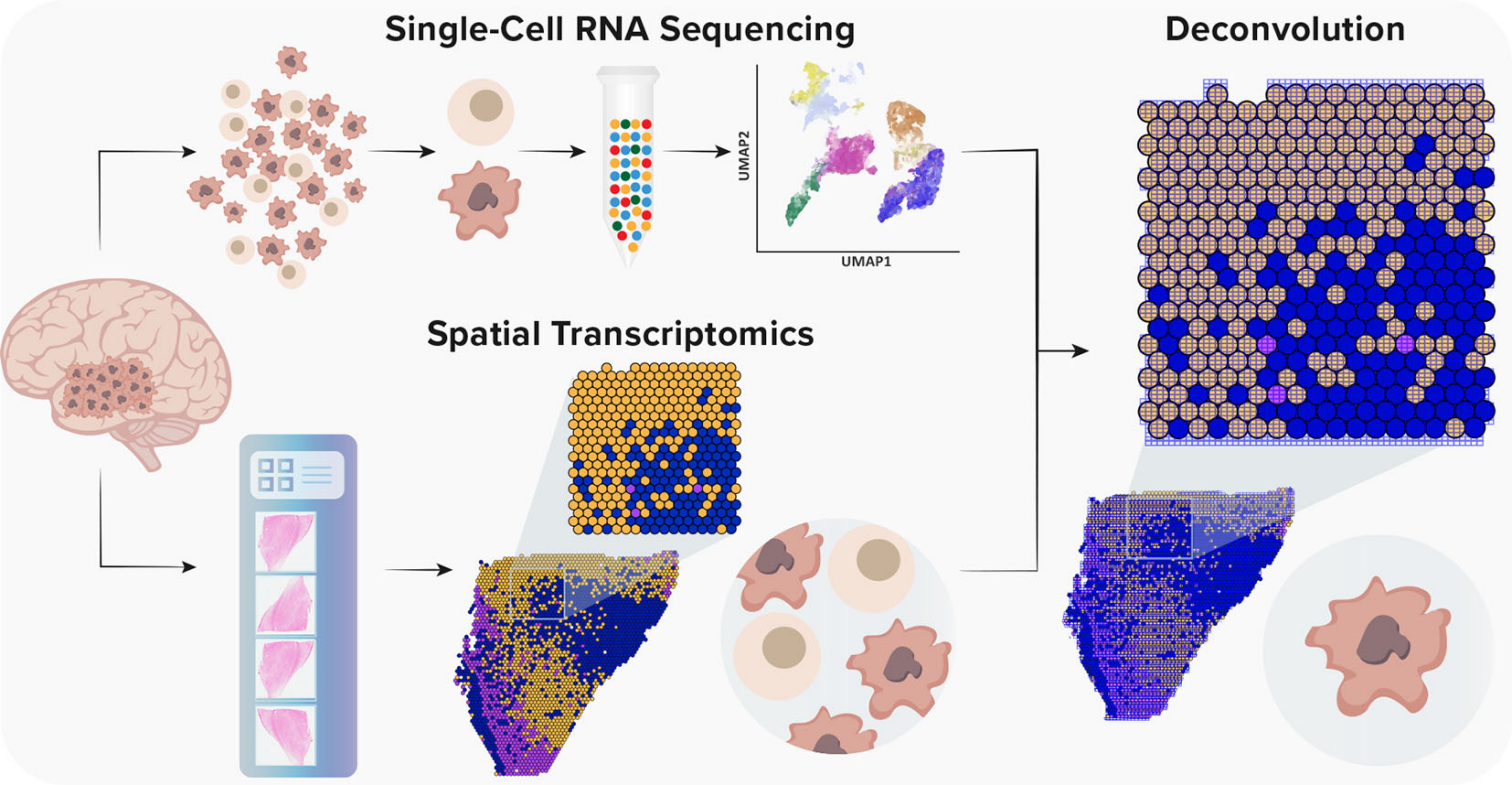
Integration (deconvolution) strategy of single-cell RNA sequencing and spatial transcriptomics data (based on the 10x Genomics protocol). Single-cell RNA sequencing provides high-throughput and high-resolution profiling of gene expression. However, it lacks spatial information due to tissue dissociation. Conversely, ST offers a spatial context without single-cell resolution. Currently, the spot diameter of ST and Visium platforms is 100 μm and 55 μm, respectively, capturing from one to 30 cells. Thus, to gain a spatial single-cell resolution, it is necessary to integrate both methods.

Although scRNA-seq should expose a detailed functional characterization of transcriptomics of a single cell and allows integration of *in vivo* states with *in vitro* models, in principle, it provides an indirect inference of cellular interactions. ^26^ However, with the recent introduction of spatial transcriptomics (ST), it is believed that spatial and functional organization are strictly related, especially in the context of a neuronal tissue. ^27^ ST is a revolutionary method that enables the characterization of cellular interactions and spatial organization of examined tissues. ^28^ Given the fact that ST is not yet at single-cell resolution, methods for the integration of scRNA-seq and ST are vital to understand the heterogeneity of GBM ( Figure 2). With the rapid development of complex algorithms and machine learning technologies, a variety of tools to integrate scRNA-seq and ST were recently developed, which enabled a precise characterization of GBM and its TME. ^29^^–^^36^

### The first layer of glioblastoma heterogeneity: bulk-RNA sequencing

**Figure 3.**
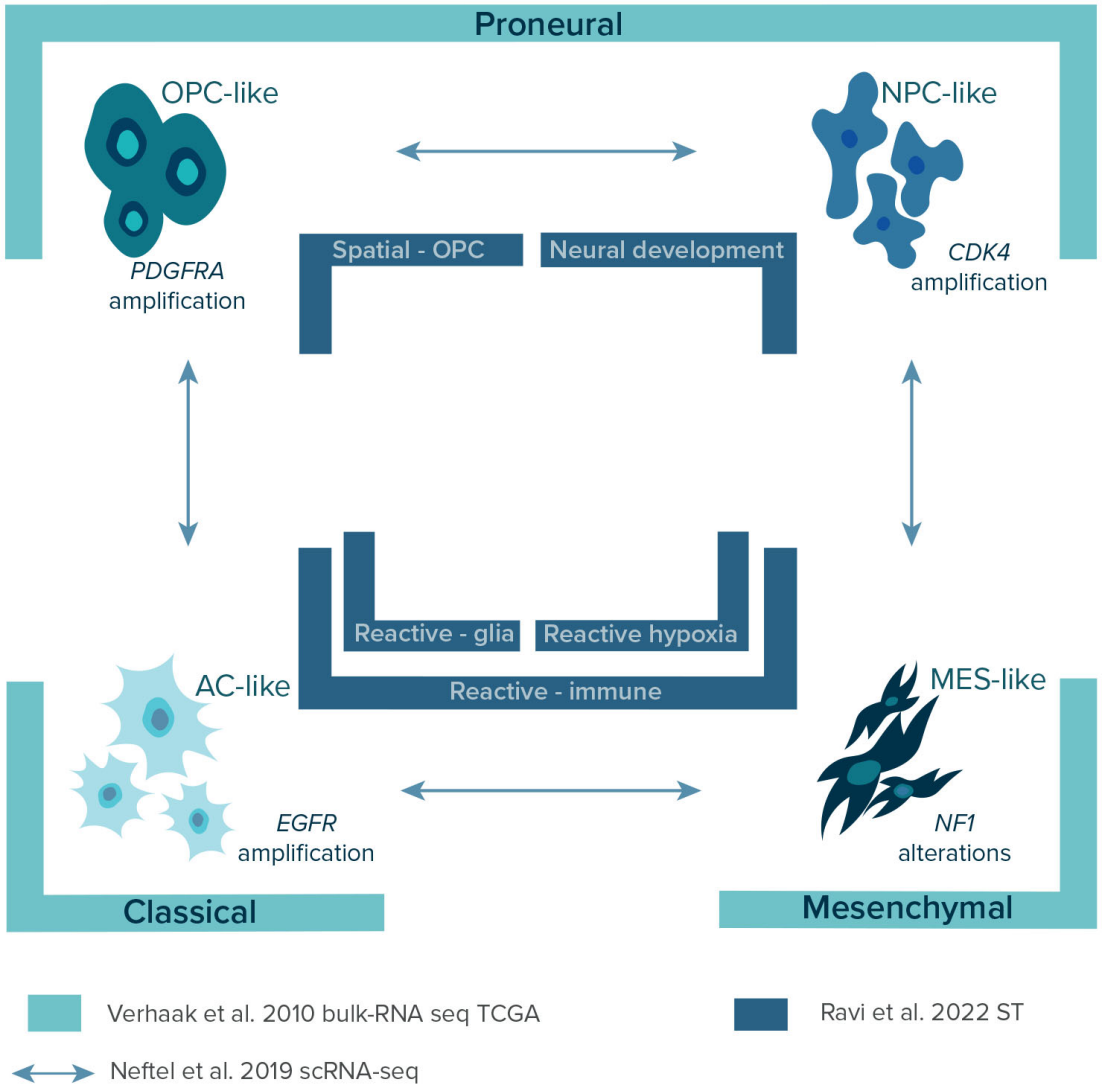
Graphical presentation of layers of GBM. Over the years, three GBM classifications were established using three different methodological approaches. This figure shows and points biological lineage of each GBM subtypes as well as differences in the nomenclature which should be unified in the future studies. Legend: AC-like – astrocyte-like; CDK4 – cyclin-dependent kinase 4; *EGFR* – epidermal growth factor receptor gene; MES-like – mesenchymal-like; *NF-1* – neurofibromatosis type 1 gene; NPC-like – neural progenitor-like; OPC-like – oligodendrocyte-progenitor-like; *PDFGRA* – platelet-derived growth factor receptor A gene; scRNA-seq – single-cell RNA sequencing; ST – spatial transcriptomics; TCGA – The Cancer Genome Atlas.

Over the years, with the constant introduction of more advanced sequencing methods and the launching of The Cancer Genome Atlas (TCGA) in 2008, a few layers of transcriptional GBM heterogeneity were discovered ( Figure 3) providing a classification of GBM subtypes. ^14^^,^^16^^,^^26^ In 2010, Verhaak *et al*., using bulk-RNA sequencing data of 200 GBM patients from TCGA, identified four distinct transcriptional subtypes of GBM: 1) classical, 2) mesenchymal, 3) proneural, and 4) neural. The results were further validated in a combined cohort of 260 GBM patients from previous studies and confirmed the association of each subtype with certain genetic events. ^37^^–^^40^ The classical subtype was highly associated with chromosome 7 amplification paired with chromosome 10 loss, *EGFR* gene alterations, and disruption of RB, Notch, and Sonic hedgehog signaling. The mesenchymal subtype was characterized by alterations in *NF1* and *PTEN* genes affecting the AKT pathway and expression of mesenchymal markers and tumor necrosis factor superfamily and NF-κB proteins. The proneural subtype was associated with various gene alterations, including *PDGFRA, IDH1, TP53, PIK3CA/PIK3R1*, and higher expression of *OLIG2, SOX, DCX, DLL3, ASCL1,* and *TCF4.* Finally, the neural subtype was associated with the expression of neuron markers *NEFL, GABRA1, SYT1,* and *SLC12A5.* However, it was later confirmed that it is not a tumor-specific subtype of GBM with a lack of gene abnormalities but resected fragments with a high contribution of normal tissues. ^15^ Next, identified subtypes were assigned to neural cell types using transcriptomic data gene sets. ^41^ The classical, mesenchymal, and proneural subtypes were associated with murine astrocytic, astroglia, and oligodendrocytic signatures, respectively. Interestingly, the frequency of each subtype can vary within the same tumor as multiple subtypes can co-exist or change over time and as a response to therapy. ^4^^,^^42^ Interestingly, the prevalence of mesenchymal, neural, and proneural subtypes is higher in males than females, while the classical subtype occurs with the same frequency. ^10^^,^^43^

### The second layer of heterogeneity: single-cell RNA sequencing

In 2019, Neftel *et al*. showed the relationships between genetic subtypes and cellular states by deconvolution of scRNA-seq and TCGA bulk data on GBMs with lineage tracing in GBM murine models. ^16^ The researchers depicted four cellular states: 1) neural progenitor-like (NPC-like), 2) oligodendrocyte-progenitor-like (OPC-like), 3) astrocyte-like (AC-like), and 4) mesenchymal-like (MES-like) corresponding to previously established TCGA signatures ( Figure 3). ^14^ As previously mentioned, these states may co-exist within the same tumor with different frequencies influenced by genetic alterations in *CDK4, PDGFRA, EGFR,* and *NF1*, which favor a particular state, respectively. Based on distinct gene expression patterns signatures can be divided into mesenchymal (MES1-like [hypoxia-independent], MES2-like [hypoxia-dependent]) and neuro-developmental (NPC1-like, NPC2-like, OPC-like, AC-like) states. ^44^^,^^45^ In general, GBM cells correspond primarily to one of the four states, however, each of the tumors contains at least two cellular states, with most tumors containing all four states. The most frequent hybrid states are AC-like/MES-like, NPC-like/OPC-like, and AC-like/OPC-like.

**Figure 4.**
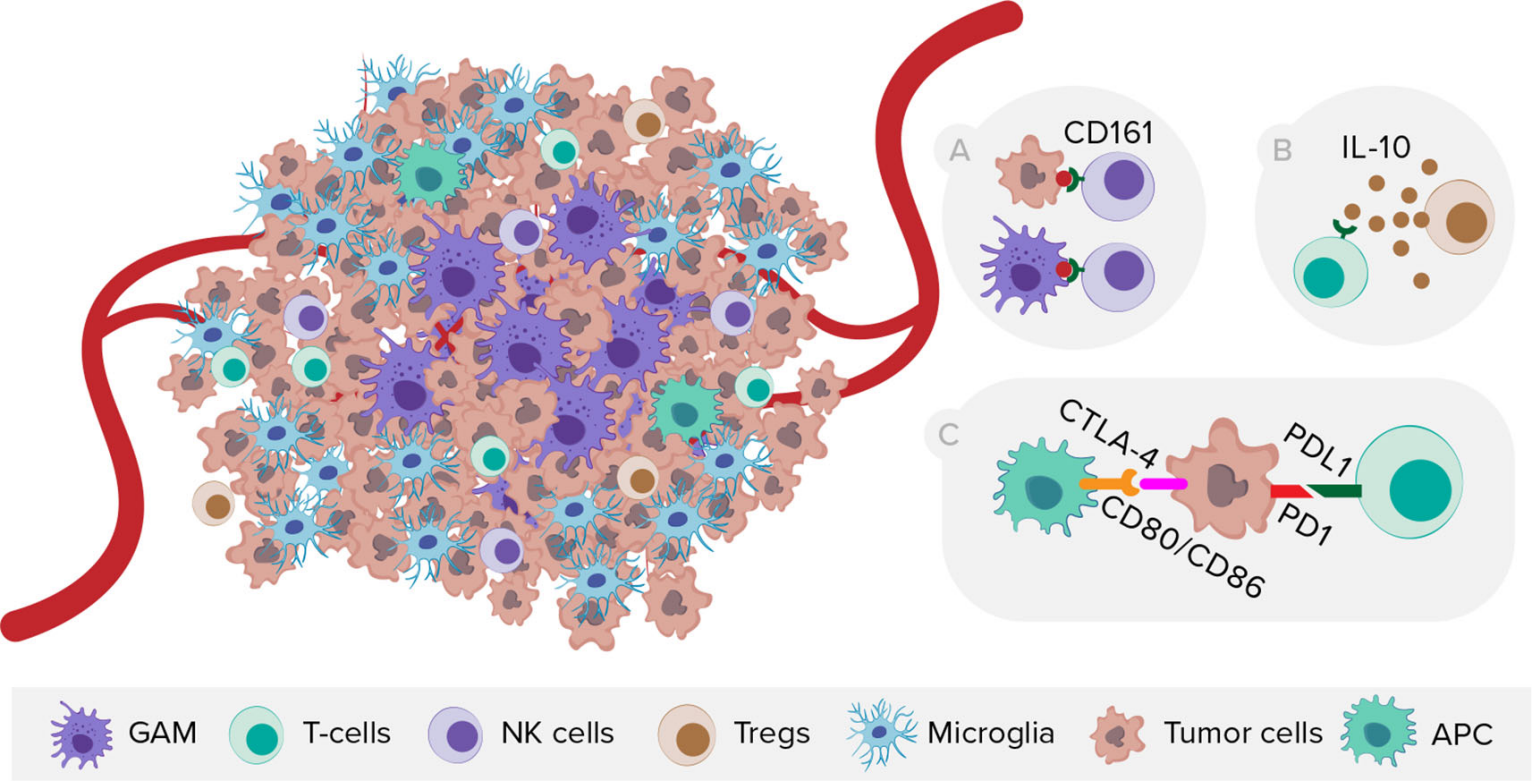
The schematic representation of GBM heterogeneity at the cellular level and selected mechanisms of immunosuppression within TME. NK cells, natural killer cells, Tregs, T regulatory cells, APC, antigen presenting cells. A) Expression of CD161 receptor by NK cells and its ligand CLEC2D by malignant and myeloid cells. B) T-cell exhaustion mediated by TME cells releasing interleukin-10. C) Expression of immune checkpoints on tumor and peritumoral myeloid cells.

On the other side, scRNA-seq provided novel insights into GBM immunology ( Figure 4), especially on the localization of GAMs within the tumor. Microglia tend to reside in the tumor periphery with the adjacent brain parenchyma, while tissue-invading monocyte-derived macrophages (MDM) are most abundant within the tumor core. ^46^^–^^48^ Moreover, the expression of immune checkpoint receptor ligands differs in myeloid cells between tumor core and peritumoral tissue. ^46^ Comparison of microglial cells from human IDH wild-type GBM and age-matched controls revealed substantially downregulated expression of microglia core genes and upregulated expression of inflammatory- ( *IFI27, IFITM3*), metabolic- ( *LPL, APOE, TREM2),* and hypoxia-associated ( *HIF1A, VEGFA*) genes. ^49^ In terms of sex-dependent differences, Ochocka *et al.* showed that microglia MHC class II-associated genes were significantly upregulated and more reactive in males than in females. ^50^ Furthermore, Pombo *et al*. investigated the evolution of functional GAM profiles across disease stages by sequencing samples from newly diagnosed and recurrent GBMs. Microglia-derived GAMs were predominant in newly diagnosed tumors, but were surpassed by more heterogeneous MDMs in the recurrent ones, especially in the hypoxic tumor environment. ^51^ Regarding T cells, Mathewson *et al*. showed that the inhibitory natural killer (NK) cells receptor CD161 is expressed in tumor-infiltrating lymphocytes, but absent in T regulatory cells (Tregs) or patient-matched peripheral blood mononuclear cells (PBMCs). Moreover, CLEC2D (CD161 ligand) was primarily expressed by malignant and myeloid cells, revealing similarities with the PD-1/PDL-1 (programmed death-1/programmed death-1 ligand) system.

### The third layer of heterogeneity: spatial transcriptomics

Local cellular interactions between tumor and cells located in TME play a major role in the adaptation of GBM and facilitate growth, infiltration, and therapy resistance, contributing to unique spatial signatures in GBM. ^52^^–^^54^ In 2022, Ravi *et al*. published an atlas of spatially resolved transcriptomics of 28 specimens (20 patients) and complemented it with spatially resolved metabolomics and proteomics. ^26^ The researchers described five spatially distinct transcriptional programs of GBM: 1) radial glia, 2) reactive-immune, 3) neural development, 4) spatial OPC, and 5) reactive-hypoxia ( Figure 3). The first two were associated with high expression of astrocyte -related genes ( *GFAP, AQP4, VIM, CD44*). Specifically, radial glia program had an increased expression of radial-glia-associated genes ( *HOPX, PTPRZ1*) and reactive-immune program had a functional enrichment of inflammation-associated genes ( *HLA-DRA, C3, CCL4, CCL3*) and interferon-γsignaling. The next two programs were associated with neuronal lineages (neurons or oligodendrocytes) and were named accordingly. The last program was associated with hypoxia-response ( *VEGFR, HMOX1, GAPDH*) and glycolytic ( *LDHA, PGK1*) genes. In order to integrate novel programs with already established bulk and single-cell classifications, spatial-weighted regression and bilateral integration of the top-scoring gene signatures were carried out, confirming an overlap between radial glia, spatial OPC, neuronal, reactive-hypoxia, and AC-, OPC-, NPC-, and MES2-like (hypoxia-dependent) states, respectively.

*Reactive-immune program*

The hybrid meta-modules described by Neftel *et al.* were associated with reactive-immune program suggesting a close functional relationship between AC- and MES-like states. ^16^ In further analysis, an imaging mass cytometry-based single-cell profiling showed significant enrichment of myeloid and lymphoid cells across hybrid regions. A substantial enrichment of tumor-associated myeloid cells and T cells was found among the reactive immune program. Moreover, the mean PD-1 protein level on T cells was increased, suggesting locally enhanced immunosuppression. These findings were supported by the enrichment of CD163+ myeloid cells, which support phagocytosis and immunosuppression. Furthermore, a previous study by Ravi *et al*. in 2022 focused on T-cell dysfunction and indicated that exhausted T cells are preferentially located within regions with mesenchymal transcriptional programs. The study revealed that the spatial and functional interaction between the myeloid and lymphoid compartment leads to an interleukin-10 mediated T cell exhaustion. ^55^ This was further confirmed that the enrichment of memory and exhausted T cells occur in the reactive-immune and reactive-hypoxia areas. ^26^

*Reactive hypoxia program*

The reactive hypoxia program is associated with histologically confirmed areas of necrosis and a high prevalence of copy-number alterations (CNAs), including focal amplification of oncogenes or losses of tumor suppressors. Spatially resolved metabolomics of reactive-hypoxia regions revealed a significant enrichment of the pentose phosphate pathway, phosphoadenylate metabolism, glycolysis, and amino sugar metabolism. ^26^ Ravi *et al*. showed that hypoxia and oxidative stress highly contribute to genomic instabilities, including various chromosomal alterations, that are driving forces in GBM resistance to therapies. Moreover, the reactive-hypoxia program is prevalent among non-cycling cells resulting in a S-phase arrest contributing to the genomic instability. ^56^ Finally, based on migratory gene-expression signatures, the effect of oxidative stress on cellular migration was explored, revealing the opposing drivers of genomic diversity, resulting in clonal evolution in GBM.

## Conclusions

In this mini-review, we showed a comprehensive overview of the GBM heterogeneity which was revealed by scRNA-seq and ST, and the urgent need to integrate both methods in future research. We discussed distinct layers of heterogeneity with regard to GBM genetics, transcriptomics, immunology, and patients’ sex. Worryingly, despite a growing number of clinical reports about the impact of patients’ sex on GBM prevalence, therapeutic outcomes, and prognosis, there are a few studies considering patients’ sex while using scRNA-seq or ST. Hence, in the future, it is crucial to distinguish patients’ sex in order to characterize potential differences which could have a major impact on the development of therapeutic agents and overcoming GBM treatment resistance.

## Data availability

There are no underlying data associated with this article.
