# Supplementary material for: Integration of single-cell RNA sequencing and spatial transcriptomics to reveal the glioblastoma heterogeneity
Source: F1000Res. 2023 Aug 9;11:1180. Originally published 2022 Oct 17. [Version 2] doi: 10.12688/f1000research.126243.2 (PMC9978243; doi:10.12688/f1000research.126243.2)
Supplement: Peer review history

# Peer review: Reviewer response for version 2

**DOI:** 10.5256/f1000research.147358.r194972

**Published:** 2023-08-10

**Authors:** Theo Mantamadiotis

**Affiliations:** 1 The University of Melbourne, Parkville, Australia

The authors have done an excellent job writing & revising this review, presenting pivotal research OMIC studies investigating the GBM tumor microenvironment and sex-specific differences.

Is the review written in accessible language?

No

Are all factual statements correct and adequately supported by citations?

Yes

Are the conclusions drawn appropriate in the context of the current research literature?

Yes

Is the topic of the review discussed comprehensively in the context of the current literature?

Yes

Reviewer Expertise:

Cancer biology, brain cancer, molecular biology, gene expression, animal models, developmental biology

I confirm that I have read this submission and believe that I have an appropriate level of expertise to confirm that it is of an acceptable scientific standard.

# Peer review: Reviewer response for version 1

**DOI:** 10.5256/f1000research.138634.r163667

**Published:** 2023-03-01

**Authors:** Georges Nemer

**Affiliations:** 1 American University of Beirut, Beirut, Beirut Governorate, Lebanon

The review by Perdyan A *et al.* recapitulates the historical classification of different types of glioblastomas. Overall, the mini-review captures all 4 pillars of heterogeneity: genetics, transcriptomics, immunogenomics, and sex. 

- In the form, there are a couple of typos and grammar mistakes as well as redundant "words" that should be corrected like "on the other side" on pages 3 and 6 and the repetition of "on the other hand" in different contexts. 
- The authors should also correct the label "in this study" on page 4, as this is a mini-review. 
- For the content, all is well-covered but it would be better if sex is integrated into the other 3 pillars of heterogeneity. 

The mini-review can be Approved after these minor corrections.

Is the review written in accessible language?

Partly

Are all factual statements correct and adequately supported by citations?

Yes

Are the conclusions drawn appropriate in the context of the current research literature?

Yes

Is the topic of the review discussed comprehensively in the context of the current literature?

Yes

Reviewer Expertise:

Transcriptomics, exome sequencing

I confirm that I have read this submission and believe that I have an appropriate level of expertise to confirm that it is of an acceptable scientific standard.

# Response

**Published:** 2023-04-22

**Authors:** Jakub Mieczkowski

In the form, there are a couple of typos and grammar mistakes as well as redundant "words" that should be corrected like "on the other side" on pages 3 and 6 and the repetition of "on the other hand" in different contexts. 

 The authors should also correct the label "in this study" on page 4, as this is a mini-review. 

**The main manuscript text was edited accordingly.**

 For the content, all is well-covered but it would be better if sex is integrated into the other 3 pillars of heterogeneity. 

**We integrated sex into one of the three pillars of heterogeneity. Unfortunately, there are only a few studies investigating sex-dependent differences. Studies on which the heterogeneity classifications were made did not take into account this matter. Hence, the purpose of this mini-review was to point out and address this gap in the field.**

# Peer review: Reviewer response for version 1

**DOI:** 10.5256/f1000research.138634.r161164

**Published:** 2023-02-17

**Authors:** Theo Mantamadiotis

**Affiliations:** 1 The University of Melbourne, Parkville, Australia

The mini-review explores the very interesting observations that male patient with GBM exhibit a higher incidence and fair worse than female patients with GBM. The authors discuss many valid biological phenomena related to the tumor microenvironment and sex-specific differences among tumor infiltrating immune cells and other cell types found in GBM.

 Although the sex-specific biology is discussed, the review is divided into sections based on technologies, rather than biology. This approach distracts from the interesting biology.

 Table 1 seems more appropriate for a technical or bioinformatic focused review, unless the authors can demonstrate that each of these approaches has discovered something relevant to the biology of sex-specific differences.

 Other parts of the manuscript which require improvement are Figure 1 legend text. The diagram and legend could be better presented. A clear explanation of what Figure 1 shows is required.

 Figure 3 is difficult to understand with all the layers of information. I don’t understand what “Chronological..” means in the figure title. Some inner dark blue sections indicate biological information (hypoxia/development), while others indicate cell types and words like “Spatial-OPC”. Can this figure be edited to fix these inconsistencies? The figure legend needs more text to explain what is shown.

 The manuscript needs editing to correct language errors. For example, in the Abstract, the words “However, despite..” are used together. “However” is redundant here, so should be deleted. The word ”exposed” as used in the abstract and with the body text, is wrong – the authors mean “revealed”. Another example is in the Conclusions section. Near the end of Conclusions, “it is warrant” is used but it's unclear what the context is.

Is the review written in accessible language?

No

Are all factual statements correct and adequately supported by citations?

Yes

Are the conclusions drawn appropriate in the context of the current research literature?

Yes

Is the topic of the review discussed comprehensively in the context of the current literature?

Yes

Reviewer Expertise:

Cancer biology, brain cancer, molecular biology, gene expression, animal models, developmental biology

I confirm that I have read this submission and believe that I have an appropriate level of expertise to confirm that it is of an acceptable scientific standard, however I have significant reservations, as outlined above.

# Response

**Published:** 2023-04-22

**Authors:** Jakub Mieczkowski

Table 1 seems more appropriate for a technical or bioinformatic focused review, unless the authors can demonstrate that each of these approaches has discovered something relevant to the biology of sex-specific differences.

**Table 1 was removed from the main text. There were no sex-specific differences mentioned in these articles.**

 Other parts of the manuscript which require improvement are Figure 1 legend text. The diagram and legend could be better presented. A clear explanation of what Figure 1 shows is required.[AP1] 

**Figure 1 was changed accordingly. A detailed description of the information present in Figure 1 is present in the introduction section. The title of Figure 1 was corrected.**

 Figure 3 is difficult to understand with all the layers of information. I don’t understand what “Chronological..” means in the figure title. Some inner dark blue sections indicate biological information (hypoxia/development), while others indicate cell types and words like “Spatial-OPC”. Can this figure be edited to fix these inconsistencies? The figure legend needs more text to explain what is shown.

**The title and description of the figure were changed accordingly. Names of specific subtypes were cited from primary publications. The goal of this article was to show and point out these differences which should be unified in future studies.**

 The manuscript needs editing to correct language errors. For example, in the Abstract, the words “However, despite..” are used together. “However” is redundant here, so should be deleted. The word ”exposed” as used in the abstract and with the body text, is wrong – the authors mean “revealed”. Another example is in the Conclusions section. Near the end of Conclusions, “it is warrant” is used but it's unclear what the context is.

**The main manuscript text was edited accordingly.**

# Peer review: Reviewer response for version 1

**DOI:** 10.5256/f1000research.138634.r153463

**Published:** 2022-10-24

**Authors:** Dieter Henrik Heiland

**Affiliations:** 1 Department of Neurosurgery, Faculty of Medicine, Medical Center, University of Freiburg, Freiburg, Germany

This is a very enjoyable overview of the various layers of omic knowledge that have been gathered over the last few years. The article is well-written and provides the information expected from a mini-review. The illustrations are well laid out and clear.

 In Fig. 2, the correct nomenclature should be taken into account. It says "reactive glia", which is incorrect and should be called "radial glia". After minimal adjustments support the indexing.

Is the review written in accessible language?

Yes

Are all factual statements correct and adequately supported by citations?

Yes

Are the conclusions drawn appropriate in the context of the current research literature?

Yes

Is the topic of the review discussed comprehensively in the context of the current literature?

Yes

Reviewer Expertise:

Single cell omics, spatially resolved multiomics, computational biology, software development, mathematical cancer-models

I confirm that I have read this submission and believe that I have an appropriate level of expertise to confirm that it is of an acceptable scientific standard.

# Response

**Published:** 2023-04-22

**Authors:** Jakub Mieczkowski

In Fig. 3 the wording “reactive glia” was used because it is an original wording used by the authors of the primary publication. The purpose of the article was to show and point out used nomenclature and provide a good background for further studies.
